# In the loop: promoter–enhancer interactions and bioinformatics

**DOI:** 10.1093/bib/bbv097

**Published:** 2015-11-19

**Authors:** Antonio Mora, Geir Kjetil Sandve, Odd Stokke Gabrielsen, Ragnhild Eskeland

**Keywords:** Chromatin loops, chromosome conformation capture, CTCF, enhancer prediction, histone modifications, promoter–enhancer interactions, SNPs, transcription factories

## Abstract

Enhancer–promoter regulation is a fundamental mechanism underlying differential transcriptional regulation. Spatial chromatin organization brings remote enhancers in contact with target promoters in *cis* to regulate gene expression. There is considerable evidence for promoter–enhancer interactions (PEIs). In the recent years, genome-wide analyses have identified signatures and mapped novel enhancers; however, being able to precisely identify their target gene(s) requires massive biological and bioinformatics efforts. In this review, we give a short overview of the chromatin landscape and transcriptional regulation. We discuss some key concepts and problems related to chromatin interaction detection technologies, and emerging knowledge from genome-wide chromatin interaction data sets. Then, we critically review different types of bioinformatics analysis methods and tools related to representation and visualization of PEI data, raw data processing and PEI prediction. Lastly, we provide specific examples of how PEIs have been used to elucidate a functional role of non-coding single-nucleotide polymorphisms. The topic is at the forefront of epigenetic research, and by highlighting some future bioinformatics challenges in the field, this review provides a comprehensive background for future PEI studies.

## Introduction

The genetic information of each individual cell in the human body is coded by the same DNA sequence. Unique cellular phenotypes are therefore accomplished through different levels of gene, RNA and protein regulation. Transcriptional regulation of gene expression is orchestrated by a series of DNA-binding proteins and protein complexes known as ‘transcription factors' (TFs), ‘co-activators', ‘co-repressors' and the RNA polymerase II (RNAPolII). TF binding mainly occurs at clusters of DNA regulatory sequences called ‘promoters' and ‘enhancers' (for a current definition of promoters and enhancers, see Boxes 1 and 2), and it is highly dependent on the differential accessibility and activity of the primary DNA sequence packed into chromatin. Generally, chromatin harbours different compaction levels, and transcriptionally active regions possess a more open structure. The openness of the chromatin also depends on the gene richness of the region [1]. Nucleosomes are the first level of chromatin organization, and are made up of an octamer of histones (H2A, H2B, H3 and H4) around which DNA is wrapped. Genome-wide mapping of histone modifications has shown a correlation of specific histone post-translational modifications with transcriptionally active (e.g. trimethylation of histone H3 lysine 4: H3K4me3) and silenced genes (e.g. H3K27me3), enhancer activity (e.g. H3 lysine 27 acetylation: H3K27ac) and heterochromatin (e.g. H3K9me3) [2–5]. Histone modifications may alter the interaction between histone and DNA or act as a binding platform for proteins or protein complexes that may mediate changes to chromatin states [6, 7].

Box 1. Promoters

The promoter is the region of DNA where initiation of transcription takes place. It includes the TSS(s) of the gene in question, but how far it extends is not precisely defined. The proximal promoter encompasses the region immediately upstream (up to a few 100 bp) from the core promoter [149], although arbitrary distance cut-offs are often seen. Best defined is the core promoter, which is the narrow DNA segment within which transcription initiates, or approximately ±40–50 bp around the TSS [150, 151].

The functional sequence elements of the core promoter, important for directing the RNAPolII to initiate RNA synthesis at a particular position, have been studied for decades. Several functional *cis*-elements have been identified such as the TATA-box and the Inr element, as well as a growing number of additional elements such as DPE, BREu and BREd, DCEs (recently reviewed in [152, 153]). These elements act together as an assembly platform for GTFs and co-activators, leading to formation of the preinitiation complex. The precise composition of core promoter *cis*-elements varies from gene to gene, probably allowing for diversity in the complexes assembled at different promoters. This diversity is also reflected in a diversity of GTFs, the use of which may vary during differentiation [154].

Global analyses of thousands of genes and their TSS have revealed that not all core promoters are equal, neither in composition, nor in function. At a first level, it is possible to classify core promoters in two main groups based on the number and clustering of TSSs and the GCcontent of the region [151, 155]. Sharp or focused promoters show a peaked distribution of TSSs with one or a few TSSs closely spaced within a few nucleotides. They are most often tissue-specific. Broad promoters show TSSs scattered over a region of 50–100 nucleotides. These promoters are usually active in many tissues, and they are often located in strong nucleosome-free regions with the first downstream nucleosome being firmly positioned. Because the sharp promoters are governed mainly by the action of various TFs, while the broad class is more dependent on chromatin accessibility, one may think of them as transcriptionally versus epigenetically controlled.

Box 2. Enhancers

Enhancers are clusters of distal DNA sequences that can increase transcription of their target gene(s) in *cis*. The distance of enhancers to their target promoter(s) vary, and in metazoans, an enhancer is placed from 100 bp to Mb away from the regulated gene on the same chromosome [114]. The enhancers can be upstream or downstream of their target genes or even placed in the gene body of another gene, and enhancer regulation can bypass other genes independently of orientation [18]. Enhancer sequences contains regulatory elements with multiple binding sites for TFs, but we have a poor understanding of a sequence code for enhancers, and although sequence conservation of enhancers has been used with some success, enhancers may have modest or no conservation [18]. Enhancer chromatin is ‘marked' by histone modifications that, in different combinations and levels, and are used to classify active (e.g. H3K4me1/H3K27ac) and intermediate or poised (e.g. H3K4me1/H3K27me3) enhancer regions [156]. Other histone modifications and histone variants have also been found to associate with enhancers (Table 3). Enhancer–promoter looping has been shown to be necessary for gene activation [157]. However, it has also been shown that opening of repressive enhancer–promoter loops lead to transcriptional activation [146, 158].

**Figure 1:**
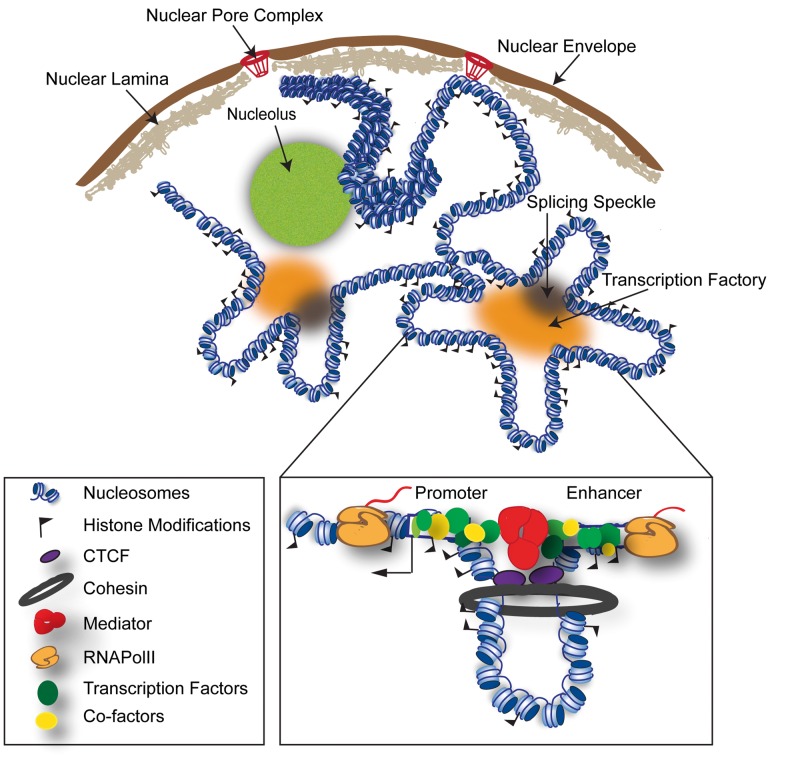
Models of chromatin organization. A diagram of different models of chromatin organization in the nuclear space. Interphase chromatin that interacts with the nuclear lamina (grey), nucleolus (green), nuclear pores (red), transcription factories (orange) and splicing speckles (black) are depicted here. Generally, lamin- and nucleolar-associated domains are transcriptionally repressed and have a more condensed chromatin, whereas chromatin that loops to the nuclear pore, transcription factories and splicing speckles are transcriptionally active and therefore have a more open chromatin structure (here, depicted as 10 nm chromatin fibre). Enhancers can activate gene expression over a distance and contain binding sites for TFs that recruit co-factors (activators or repressors). A promoter–enhancer looping mechanism mediated by cohesin (brown), CTCF (purple) and the mediator complex (red) that brings the enhancer into close proximity to its target promoter are presented in the enlarged box. The enhancer and promoter are marked with white boxes, and the transcription start site of the transcribed target gene is annotated with an arrow. TFs (green) and co-factors (yellow) bind the enhancer and are brought close to the basal transcription machinery at the promoter. RNAPolII (orange) transcribes pre-mRNA from the target gene and eRNA from the enhancer. Some of these models may co-exist for different PEIs; however, there are also other models that we could not show. A colour version of this figure is available online at BIB online: https://academic.oup.com/bib.

The activity of an enhancer is often restricted to a cell type or tissue, related to developmental or environmental conditions. Approximately 80% of all characterized mouse enhancers show tissue-specific expression [159]. RNAPolII has been found to overlap with enhancers (Figure 1) [160]. eRNAs are generally short non-coding RNAs that are bidirectionally transcribed from active enhancers and therefore used to classify enhancer activity [76, 161]. The presence of eRNAs has been suggested to precede the activation of nearby genes and eRNAs may have a role in enhancing or stabilising PEIs [27] or promoting elongation [162].

The mammalian genome contains around 23 000 genes, and an estimate of 1 million enhancers, which suggest that there is about four enhancers per gene per cell type [20]. Different enhancers may regulate the same target gene dependent on developmental stage or cell type. The combined role of groups of enhancers has also been studied. LCRs are a group of multiple enhancers responsible for coordination of temporal expression of linked genes on differentiation or during development (examples are LCR-regulating globin genes, or Global Control Region regulating *HOXD* genes) [163]. High levels of subunits of the Mediator complex (Med1) mark a group of putative strong enhancers spanning from a few kb to 50 kb, termed ‘super-enhancers' [120, 164]. The beta-globin LCR has been defined as a ‘super-enhancer' in human K562 cells [120].

Gene regulation in *cis* because of binding of proteins at specific DNA elements far from their target genes has long been recognized as fundamental in higher eukaryotes [8–11]. However, the idea that chromatin looping was responsible for long-range regulation in *cis* has become widely accepted only after the development of the ‘Chromosome Conformation Capture' (3C) technology by Dekker and co-workers [12, 13]. Promoter–enhancer interactions (PEIs) represent a subset of chromatin interactions that are central to the currently accepted model of transcriptional regulation. There is increasing support for PEIs being necessary for transcriptional regulation of an enhancer’s target gene. For example, there is evidence that the expression of a target gene is affected by gain or loss of competing promoters, lack of some PEI-associated proteins and addition of PEI-disrupting insulators [14], as well as evidence that chromatin interactions are highly associated to gene co-expression rates [15]. However, important challenges remain, such as finding the mechanisms that mediate PEIs, the building of high-resolution PEI maps in different cell types and the identification of functional interactions.

There are several recent reviews reporting on different aspects of PEIs [14, 16–23]. However, there is a paramount need for a review of the wide spectrum of bioinformatics methods developed in the field. In this article, we first give a brief description of the chromatin complexity landscape, chromatin looping mechanisms and limitations of the experimental methods. Then, we critically review associated bioinformatics issues such as data representation and visualization, raw data processing, and PEI prediction, while we highlight some of the future challenges in the field.

## Biological background

### Our knowledge of the chromatin landscape is becoming increasingly more complex

Chromatin interactions span different types of regulatory elements. In addition to PEIs, promoter–promoter looping or enhancer–enhancer looping [24], polycomb response element–promoter interactions (in *Drosophila*), insulator–insulator interactions and 5′-3′ gene looping have been mapped [25]. Exons can interact with both enhancers and promoters, and this occurs at DNase I hypersensitive sites (DHS) [26]. Moreover, the loci of a class of long non-coding RNAs called activating RNAs have been reported to behave in an enhancer-like manner to form DNA loops with their neighbouring genes through the Mediator complex [27]. Distinguishing PEIs from all other chromatin interactions is a first challenge that demands both accurate enhancer detection methods and comprehensive genome annotation efforts.

Current research has also shown at least two different types of chromatin interactions. Besides transcription-dependent chromatin interactions of promoters and enhancers, several studies have shown that the spatial configuration of the chromatin may be established and not affected by transcription [28, 29]. Understanding the differences between a transcription-dependent interaction and a transcription-independent interaction is a challenge with significant consequences for the way that we correlate PEI data to transcription data.

Finally, as our understanding of enhancers and promoters increases, their difference appears to diminish. Enhancers are transcribed, assemble general transcription factors (GTFs), RNAPolII and elongation factors [16, 30, 31]. Enhancers and promoters appear to have a common architecture with similar frequencies of core promoter *cis*-elements and highly positioned flanking nucleosomes [32]. Some intragenic enhancers have been found to act as alternative promoters [33], while some promoters have also been shown to share properties with insulators, making the distinction of regulatory elements in the chromatin landscape even more blurry [34]. It has been suggested that the best distinction between enhancers and promoters are the differences in the produced RNAs, such as transcript stability, level, length, polyadenylation and splicing [30, 32], with messenger RNAs (mRNAs) being longer and more stable than enhancer RNAs (eRNAs). New unified models of the transcriptional *milieu* have been suggested, where the promoter–enhancer distinction has been eliminated [35]. This would imply a reformulation of the PEI concept.

### There are several models of chromatin looping, and some of these biological phenomena may co-exist

In recent years, the discussion in the field has been focused on the nature of chromatin loops and the forces that generate and stabilize them [19]. One model suggests that rigid ‘active chromatin hubs' (ACH) are formed at regulatory elements in the form of complexes including TFs, transcription machinery proteins and special ‘communication' proteins, which recruit promoters to the ACH [19, 36].

This model is challenged by a second model that suggests that ACHs are better described as ‘active nuclear compartments', where chromatin fibres with regulatory sequences get trapped in space with the aid of ring-like cohesin complexes (Figure 1) [19]. It has been reported that cohesin co-localizes with Mediator complexes at enhancers and promoters, where it has a role in mediating PEIs [37]. Furthermore, cohesin is recruited to sites bound by the insulator protein CTCF (CCCTC-binding factor), even though some sites are unique for cohesin [38, 39].

A third model suggests that insulator elements put enhancers and promoters in close contact [19]. Several mechanisms of insulator function have been proposed. According to the ‘topological loop model', insulators divide the genome in independent loops corresponding to topological domains, which favour the contact between promoters and enhancers [40]. Polymer simulations have supported the existence of both insulation and facilitation processes behind PEIs [41]. CTCF has been found to bind and link two insulators that indirectly put promoters and enhancers in close proximity [13, 42]. However, CTCF can also directly bind to an enhancer and a target promoter and link them [43]. Another scenario describes CTCF binding to an insulator and a promoter [44, 45].

A fourth model suggests that genes and regulatory elements could be repositioned to nuclear compartments such as splicing speckles and transcription factories (Figure 1) [46, 47]. Splicing speckles are structures enriched in the Serine/arginine-rich (SR) splicing proteins. Transcription factories are discrete polymerase and transcription foci in the nucleus [48]. Current models depict the factory as a polymorphic compartment with a ‘diameter' of ∼60–200 nm [35], where RNAPolII molecules remain stationary in the ‘surface', while the core is rich in proteins such as TFs, co-activators, chromatin remodellers, histone modifiers, RNA helicases, and splicing factors. Chromatin loops can bind to RNAPolII molecules on the surface of these factories, and can do it multiple times and become organized around them in complex shapes, such as ‘rosettes' [35, 49]. Many transcription factories are specialized, in the sense that they are enriched in a given TF, and several genes regulated by this TF are repositioned to this factory and transcribed [48]. Examples of genes that move to transcription factories include interleukin-coding genes and their regulatory regions, globin locus and its Locus Control Region (LCR), the human pituitary growth hormone gene and its LCR, and Hox genes [35].

In a fifth model, genes are looping to or away from nuclear landmarks such as the nuclear lamina, heterochromatin, nucleolus and nuclear pore complexes (NPCs) (Figure 1) [50]. The nuclear envelope is made up of a double lipid membrane perforated by NPCs that allow import and export into the nucleus. A dense mesh of nuclear lamina proteins coats the inside of the nuclear membrane. Lamin-associated chromatin domains are generally gene poor, late replicating and rich in repressive histone marks, whereas NPC-interacting chromatin sites comprise genes that are modestly transcribed [51]. Transcriptionally inactive chromatin is also found at the periphery of the nucleolus [52]. The ‘stemness' genes (*Oct4*, *Klf4* and *Nanog*) that are important for pluripotency in embryonic stem cells (ESCs) move to the periphery on neuronal differentiation concomitant with gene silencing [53]. Moreover, polycomb-repressed *Hox* genes can fold into chromatin domains (termed polycomb bodies in flies) [54].

Gene regulation may comprise all five models. Rieder *et al.* have found that both transcription factories and splicing speckles could have a role in PEIs [55]. Rao *et al.* report that most chromatin loops are anchored by CTCF (86%) and the cohesin subunits RAD21 (86%) and SMC3 (87%) (and to a lesser extent, by ZNF143 and YY1), supporting the role of CTCF and cohesin in PEI regulation [56]. What is the cause or consequence for transcriptional regulation in these models is still uncertain. However, all models point to the necessity of modelling of spatially co-localized groups of genomic regions (and not only linear sequences) to understand transcriptional regulation.

### The limitations of the existing experimental methods are a source of bioinformatics challenges

Insights into PEIs have been obtained by different techniques. Imaging techniques, mainly with Fluorescence *In Situ* Hybridization (FISH), have extensively been used to study three-dimensional (3D) folding of chromosomes, and positioning of gene loci and chromosomes in the nuclear space [57]. Immuno-FISH has revealed that genes are associated with specific subnuclear structures, such as transcription factories and the nuclear periphery [58]. Moreover, this method has shown that active gene loci can loop out of their chromosome territory [59, 60], and have been used to characterize PEIs for gene loci such as *Hoxb13*, *Shh* and the olfactory receptor [61–63]. However, FISH can only be used for a limited number of DNA loci at a time, and not in a high-throughput manner, and its spatial resolution is limited.

Development of the family of ‘3C techniques' has made it possible to study chromatin organization on a genome-wide scale [64]. These methods include 3C [12], 4C (circular 3C) [65], 5C (3C-carbon-copy) [66], Chromatin Interaction Analysis with Paired-End-Tag sequencing (ChIA-PET) [67] and Hi-C (Genome-wide 3C) [68]. 3C and 4C analysis require choosing a target locus (a ‘view-point') to map interaction with another locus or genome-wide, respectively. 5C probes all interactions with multiple selected viewpoints within a confined genomic region (typically 1 Mb), while Hi-C allows unbiased genome-wide analysis of chromatin interactions with resolutions that have been improving up to 1 kbp [56]. ChIA-PET is a combination of 3C, chromatin immuneprecipitation (ChIP) and paired-end tag sequencing that allows the genome-wide identification of potential interacting loci bound by a given TF, RNAPolII, or enriched by histone modifications [69]. Capture Hi-C (CHi-C) is a recent method to map interactions of promoters with distal elements by using solution hybridisation selection to enrich for promoters and their contact regions in Hi-C libraries [165].

3C-based methods generally determine the frequency of interactions in a population of cells for which a pair of loci is close enough in space to become cross-linked (that is, approximately in the 10–100 nm range), at the time that the cells were fixed [17]. However, spatial proximity alone does not imply a functional interaction between loci. Dekker *et al.* suggest at least four different types of processes leading to spatial proximity: a direct functional interaction, a baseline interaction (random collisions), a bystander interaction (DNA close to a direct interaction will be also close as a consequence of the former) and an interaction with the same nuclear structure (such as transcription factories) [17]. Recent criticism has been directed against all ‘formaldehyde cross-linking'-based technologies such as ChIP-seq and 3C because of the fact that cross-linking efficiency varies between different proteins, or between proteins and DNA, and the fixation may trigger DNA damage response, and that the chromatin contacts are reflecting the cell populations and not individual cells [64, 70, 71]. Improvements to the Hi-C protocol, such as increasing the sequencing depth and decreasing the presence of random ligations have been put forward [64].

A recent study has systematically compared FISH and 5C measurements of the *HoxD* locus in ESCs and found that although there were several agreements between the data sets, there were conflicting observations where 5C maps display a higher interaction frequency for a region that by FISH was observed as decompacted [62]. This discrepancy raises the issue of whether there is a need of reassessment of current bioinformatics analysis methods for 3C-based data. Furthermore, both FISH and 3C methods study chromatin in cross-linked cells and do not address the dynamic movement of specific loci. A major frontier in the field will be to track the movement of individual gene loci within the nucleus, and recent development of the Clustered Regularly Interspaced Short Palindromic Repeats-associated protein-9 nuclease (CRISPR/Cas9) and chromosomal tag systems hold promise to become powerful technologies for live-cell imaging [72–74].

Another approach is the Cap Analysis of Gene Expression (CAGE) [75]. It has been shown that both enhancers and genes that are in contact are often transcribed [35]. Moreover, eRNA levels correlate with mRNA levels at nearby genes and, at the same time, eRNA transcription requires the presence of the promoter [14, 76]. The Functional Annotation Of The Mammalian Genome Phase 5 (FANTOM5) consortium used CAGE to detect active enhancers in over 800 samples spanning most human cell types and tissues [77]. FANTOM5’s pipeline starts by building a CAGE-based transcription start site (TSS) atlas, then making a distinction between eRNAs and mRNAs to detect active enhancers. To detect PEIs, the authors examined Pearson correlations in expression between all possible promoter–enhancer pairs within 500 kbp. As a result, 40% of the active enhancers were associated with the nearest RefSeq (an NCBI Reference Sequence Database) TSS, while 64% of the enhancers had at least one correlated RefSeq TSS within 500 kbp [77]. This is in contrast to the results of Sanyal *et al.* [78], who reported that only 7% of the loops are established with the nearest gene measured by 5C in 1% of the human genome (ENCODE pilot project regions) in three different cell types (GM12878, K562 and HeLa-S3 cells).

Recently, FANTOM5 Phase2 has published a CAGE analysis of different time courses in 19 human and 14 mouse cell lines [79]. The results show that 13% of human enhancers and 20% of mouse enhancers significantly changed expression over time in at least one time course. More interestingly, promoters and enhancers are not co-expressed over time, as eRNA transcription is generally an early event and rapidly returns to a baseline [79]. This observation has consequences for eRNA-based prediction of PEIs, as PEIs may be more stable than the rapid eRNA transcription, and therefore other parameters must be included. Also, correlating expression of enhancers with nearby genes is limiting, and potential long-range PEIs are left out.

### The estimated size of the Promoter–Enhancer Interactome depends on cell type, experimental method and data processing methodology

Some of the first systematic genome-wide studies of chromatin interactions were performed using the ChIA-PET technology. Chepelev *et al.* used ChIA-PET with an H3K4me2 antibody in T cells to identify 6520 long-distance (>20 kb) chromatin interactions [80]. In this data set, the authors identified 2067 potential enhancers interacting with 1619 promoters, generating a network of 2373 promoter–enhancer, 478 enhancer–enhancer and 3669 promoter–promoter interactions. In total, 9% of these enhancers were shown to interact with multiple promoters (several genes controlled by the same enhancer), while 25% of all promoters interact with more than one enhancer [80, 81]. Li and co-workers performed a similar study using ChIA-PET to map long-range interactions with RNAPolII in five cultured human cell lines (MCF7, K562, HeLa, HCT116 and NB4). Their network contained 938 promoter–gene internal regions, 6530 promoter–enhancer, 4106 enhancer–enhancer and 8282 promoter–promoter interactions across the different cell lines [24]. In another similar work, DeMare *et al.* built a cohesin-associated interaction map in mouse limb bud [82]. In this study, 2264 cohesin (SMC1A)-based interactions were found, where 1491 were co-occupied by CTCF, showing that these two proteins are involved in tissue-specific PEIs. The authors also observed cohesin-associated interactions that are maintained across tissues, where promoters become activated on differentiation [82].

Hi-C started as a low-resolution technology, which made it more useful to study higher-order chromatin models rather than specific PEIs [68]. However, this has been changing rapidly. Jin *et al.* performed a high-resolution Hi-C study (5–10 kbp resolution) in human fibroblasts [82]. The study reported over a million long-range interactions and also showed that, for Tumor Necrosis Factor (TNF)-alpha signalling, TNF-alpha responsive enhancers are in contact with promoters even before signalling starts, suggesting that the chromatin interaction landscape may be stable. In the most recent high-resolution genome-wide study [56], Rao and colleagues have generated a genome-wide *in situ* Hi-C study for nine cell lines in human and mouse, with a kilo base resolution (GM12878 cells were mapped at a 950 bp resolution in two biological replicates, while the other eight cell types were mapped at 5 kb resolution but only one biological replicate each). Under their experimental settings, proximity ligation was performed in intact nuclei, to reduce the number of spurious contacts that may happen in solution. The chromatin interaction landscape shown by the *in situ* Hi-C study includes approximately 10 000 chromatin loops, which are enriched in PEIs, that is, 30% of the peaks include an annotated promoter and a predicted enhancer (versus 7% expected by chance). In addition, the set of genes participating in PEIs had higher expression than the set of genes not associated to a loop. Loops were also found to be mainly short, with only 2% of the peaks corresponding to loops that are >2 Mb long [56]. A recent technology called HiCap reports a network of 15 905 promoters and 71 984 distal regions in mouse ESCs [84]. The fact that different high-throughput studies report different numbers of chromatin loops represents a challenge for both PEI data processing methods and PEI predictions.

## Bioinformatics challenges

### What is the best way to represent PEI data?

**Figure 2:**
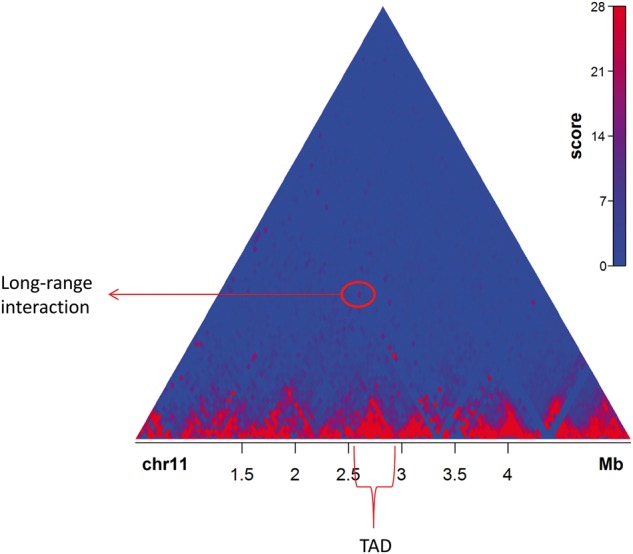
An example of a Hi-C contact map. Hi-C contact map of a segment of mouse chromosome 11, generated using Sushi [90] from Dixon *et al.* [85] data. A TAD and a long-range interaction between two loci are annotated. A colour version of this figure is available online at BIB online: https://academic.oup.com/bib.

**Figure 3:**
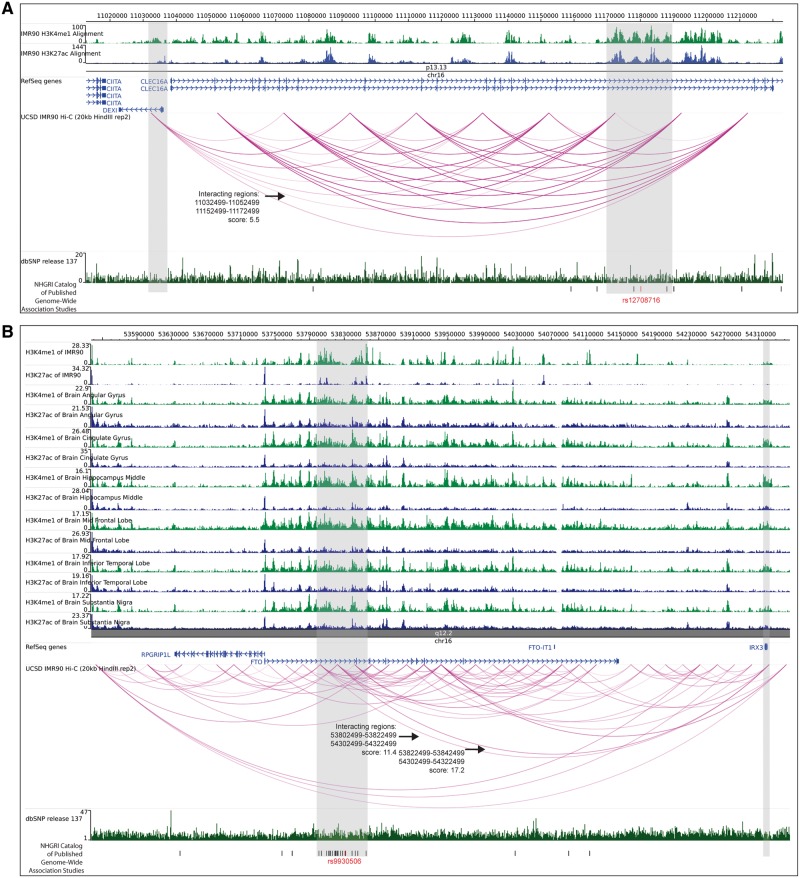
Long-range interactions functionally connect disease-associated SNPs with disease candidate genes. (**A**) Physical proximity between *DEXI* gene locus and autoimmune disease-associated SNPs in the intron of *CLEC16A*. Hi-C data from human foetal lung (IMR-90) cells (from the Ren lab, [85]) show interactions between *CLEC16A* intron 19 and the DEXI locus. The enhancer marks in IMR-90 cells for H3K4me1 and H3K27ac are shown in green and blue, respectively, and the filter threshold for the Hi-C data was set to 5. SNPs in the region are in black, and the eQTL SNP rs12708716 is marked in red. The arc (pink) for interacting regions (grey) is highlighted with an arrow. (**B**) Long-range interactions links obesity-associated variants in *FTO* with the *IRX3* locus. Hi-C data in human foetal lung (IMR-90) cells show interactions between the first intron of *FTO* with *IRX3*. The tracks for H3K4me1 and H3K27ac are shown in green and blue from IMR-90 cells and different human brain tissues from the NIH Roadmap Epigenomics Mapping Consortium. The filter threshold for the Hi-C data was set to 10. SNPs in the region are in black, and the BMI-associated SNP rs9930506 is marked in red. Arcs (pink) for interacting regions (grey) are highlighted with arrows. These public data sets are available and visualized with the WashU EpiGenome Browser (http://epigenomegateway.wustl.edu/browser/). dbSNP release 137 is shown in dark green, and the The National Human Genome Research Institute (NHGRI) Catalogues of GWAS are visualized in UCSC browser (http://genome-euro.ucsc.edu) [166]. A colour version of this figure is available online at BIB online: https://academic.oup.com/bib.

Chromatin interaction data have been traditionally represented either as a bi-dimensional heatmap or as a linear graph with ordered nodes. The «heatmap» view has been especially useful for the visual detection of regions enriched on intra-region interactions (so-called «compartments» or «TADs») (Figure 2). The alternative is the use of «arcs» that connect two interacting sites (Figure 3). This view is mainly useful to give a sense of interactions under the network analysis framework. Heatmap and arc views are related to contact maps generated either as matrices or as lists of edges, respectively.

A Hi-C contact map displays all chromatin interactions found in an experiment. Firstly, the genome is partitioned into bins of fixed size. Then, a contact matrix is generated, where every cell corresponds to the number of contacts between the associated pair of loci. Such a matrix can be visualized as a ‘heatmap', where pixel intensity correlates to the likelihood of such an interaction. It can be easily observed that loci usually interact more often with nearby loci (Figure 2). Heatmaps display triangular regions where all loci seem to heavily interact with each other, while poorly interacting with loci outside this region; therefore, a cell’s genome has been suggested to be divided into domains enriched on intra-domain chromatin interactions, called topological associating domains (TADs) (Figure 2) [85, 86]. TADs have been characterized by having boundaries enriched in CTCF motifs and Short Interspersed Nuclear Elements (SINE) retrotransposons, as well as transfer RNAs. Loss of CTCF has been shown to decrease intra-TAD interactions and increase inter-TAD interactions [87]. TADs have been described to have a median size of 400–500 kb, and their number has been proposed to be over 2000, covering 90% of the genome [17]. Recently, improved resolution has allowed a finer detection of domain boundaries and therefore smaller domains have appeared. Rao *et al.* have identified ‘contact domains' with a median size of 185 kb [56]. However, in both cases (TADs and contact domains), CTCF-binding sites are found at the boundaries, even though CTCF does also bind inside TADs [17, 56].

There are several reasons why the matrix has been the dominating conceptual representation for chromatin interaction data, here discussed in terms of Hi-C. Firstly, many of the early analyses, such as the detection of global compartments, fit naturally with principal component analysis on a matrix representation. Secondly, strong local interactions turn up along the diagonal, while more-specific, strong, long-range interactions turn up as deviating (high valued) pixels away from the diagonal (Figure 2). Similarly, TAD structure may be seen as demarcated triangles along the diagonal (Figure 2). Finally, the early Hi-C studies were of a relatively low resolution (e.g. 1 Mb), meaning that full interaction matrices could be conveniently represented and visualized. With the recent developments of Hi-C technology, where resolution is approaching that of single restriction fragments and focus is shifting towards individual specific interactions, we expect the representation of data to be rethought, and we argue that the graph may be a more suited metaphor. Dense graphs, such as those resulting from the early Hi-C experiments, are efficiently represented as an adjacency matrix; however, the size of an adjacency matrix grows proportionally with the square of the number of vertices (bins), and, therefore, an adjacency list would be much more efficient, typically several orders of magnitude smaller. Similar reductions of storage requirement could also be achieved by using some form of sparse encoding of an adjacency matrix, but that would be an unnecessarily complex approach to achieve essentially the same thing. In addition to concerns around storage efficiency, the shift in focus from global organizational features to specific local interactions that is allowed by the increased resolution and accuracy also bears better together with an adjacency list. In the context of PEIs, the ability to directly read out the list of interactions associated with a given promoter or enhancer is convenient.

There is no widely established format for representing chromatin interaction data, and there has been a tendency for every major study to provide their data in their own *ad hoc* format (see [68] versus [56], as an example). A formalized standard for representing interaction data in the form of an adjacency list has been provided by the GTrack format. This represents bins (fragments) as genomic coordinates associated with an ID, and based on this represents interactions as a list of IDs for the neighbours of a vertex [88]. Another partly formalized representation is an extension of the ‘bed' format, termed «bedpe» (Browser Extensible Data Paired-End). This format consists of ten columns, including the chromosome, starting position and ending position of each interacting region, a score, and others, and it was initially developed in the framework of the BEDTools project [89]. The «Sushi» R package [90] makes use of an extended version of this format, adding an additional column to specify the data set (and, therefore, the corresponding colour in an arc view), while relating the score to the height of the arcs.

Currently, we also expect the development of standard file formats that are prepared to store information regarding the hierarchical nature of genomic elements (chromosomes, compartments, TADs, loops, promoters and enhancers, epigenetic marks and more), as well as their dynamic behaviour, given that time-course studies will reduce current data to static time points and will open a new dimension for PEI studies.

### How to visualize PEI data?

The «WashU Epigenome Browser» [91] is one of the main examples of tools to visualize and navigate PEI data (Figure 3). This browser allows visual exploration of contact maps both as matrices/heatmaps and as arcs, while also containing a rich database of annotated data sets. A few other repositories that contain chromatin interaction data include ‘GEO' [92], ‘Array Express' [93], and the ‘3DGD database’ [94, 167]. Different labs have generated their own browsers to visualize the data sets they have produced. In this category, we find: the «Hi-C browser» by the Ren Lab [95], and «Juicebox» from Lieberman-Aiden's lab [56]. WashU and Hi-C’s browsers are web-based tools, while Juicebox is a stand-alone application. It is also possible to find libraries that allow more control to programmers. «Circos» [96] is an increasingly popular library for circular plots, originally written in Perl, which has versions in R, such as ‘Rcircos' [97]. Also in R, it is possible to find libraries such as the abovementioned «Sushi» [90].

Besides all of the abovementioned options, multiple challenges remain for PEI visualization software. More efforts are needed regarding annotation of interacting regions, as well as visualization of PEIs in multiple scales (short- and long-range interactions). Circular diagrams, such as those in ‘Circos', are one attempt to visualize interactions happening in multiple scales, from kbp to Mbp to inter-chromosomal, but results are only clear for small data sets. For this reason, we argue that better solutions are still needed.

### How should interaction raw data be processed?

Post-processing of chromatin interaction data from the 3C family of methods is an active area of research, and there are many post-processing methodologies and software tools available, but no acknowledged standard (see [98], or Table 1 for some selected tools).

**Table 1:** Processing software for 3C-type of data

Software	Authors	Description
r3Cseq	Thongjuea *et al.*, 2013 [129]	R package. It uses BAM alignment files as input, and performs aligned reads counting, read count normalization, statistical analysis of interactions and data visualization or data export of contacting regions
HiTC	Servant, 2012 [130]	R package. It allows the use of both 5C and Hi-C data and offers quality control, normalization and visualization of heatmaps
fourSig	Williams, 2014 [131]	R package. Includes fragment filtering options
Basic4Cseq	Walter, 2014 [132]	R package. Also includes fragment filtering options, and provides more sophisticated visualization options
My5C	Lajoie, 2009 [108]	(http://my5c.umassmed.edu/welcome/welcome.php)
HOMER suite	Heinz, 2010 [133]	(http://homer.salk.edu/homer/interactions/index.html)
Hi-Browse	Paulsen, 2014 [134]	(https://hyperbrowser.uio.no/3d/)

Post-processing of chromatin interaction data is needed for several reasons. One of the main reasons is that chromatin is locally dynamic and its 3D coordinates are highly variable in a population of cells, and, therefore, the probability of contact between two loci is never zero. In 3C methods, interaction frequencies have been found to decrease exponentially with increasing distance. Therefore, meaningful interactions are detected as local peaks over a decaying baseline of interactions [17]. In 5C methods, the entire data set is usually used to generate an estimate of the baseline interaction frequency for each locus, and the loop interactions are detected as peaks higher than the baseline at a given *P*-value and false discovery rate [17].

Most bioinformatics efforts have been focused on developing normalization methodologies and determination of significant interactions of Hi-C data. The first reason is to identify and account for the effect of the random (non-functional) polymer looping of the DNA versus functional chromatin interactions [99–101]. Secondly, to remove biases because of technical characteristics of the experiment, such as cross-linking preference and fragment length [102–105]. It has been stated that formaldehyde fixation introduces a fragment length bias for sizes <800 bp. That is, when fragments are <800 bp, longer restriction fragments are more likely to be cross-linked [104]. Ligation efficiency has been shown to be optimal for restriction fragment pairs of similar sizes, given that differences in size may add some distance between fragment's ends, leading to a decrease of the ligation probability [102]. Finally, there are sequencing-related biases, which are constantly being addressed through sequencing protocol modifications [106]. Fragments with too low or too high GC-content, as well as highly repetitive sequences, are under-represented among interaction reads [102]. The choice of a restriction enzyme (and, therefore, the size of the fragments), as well as the depth of sequencing, may determine if an interaction is detected or not. Other aspects that need to be kept in mind are: (a) The measurements in a static time-point (ignoring possible dynamic behaviours), (b) the fact that proximity (or interaction) is not equal to biological significance, (c) the fact that ligation probabilities are not just a function of proximity but also of reactivity, crowding and other variables and (d) that the nature of the method only allows detection of pairwise (and not multiple) interactions [106].

Some of the most popular pipelines developed to normalize Hi-C data are from Yaffe and Tanay [102] and Imakaev *et al.* [103]. Both methods are available as software tools, as shown in Table 2. Ay and Noble have recently reviewed the different algorithms used by several Hi-C tools, including both sequencing and normalization issues [23]. The authors group all normalization methods into explicit-factor [102], matrix balancing [56, 103, 104] and joint correction methods [83], with some software tools providing more than one method. Ay *et al.* have also introduced ‘Fit-Hi-C' [107], a method to assess the statistical significance of chromatin interactions that includes both the random polymer looping effect and the abovementioned observed biases in Hi-C data sets.

**Table 2:** Normalization pipelines for Hi-C data

Software	Authors	Reference originally applied	Software source
Hiclib	Mirny Lab	Lieberman-Aiden, 2009	http://mirnylab.bitbucket.org/hiclib/
Hicpipe	Tanay Lab	Lieberman-Aiden, 2009	http://compgenomics.weizmann.ac.il/tanay/?page_id=283
scell_hicpipe	Tanay Lab	Nagano, 2013	http://compgenomics.weizmann.ac.il/tanay/?page_id=580
Juicebox	Lieberman-Aiden Lab	Rao, 2014	http://www.aidenlab.org/juicebox/

Yaffe and Tanay generated a normalization pipeline that corrects for fragment length, GC content and mappability, following a maximum likelihood optimization procedure. This method has been applied to several Hi-C studies [100, 135]; however, it does rely on prior knowledge about the abovementioned sources of bias. Imakaev *et al.* developed a computationally less expensive iterative correction procedure, which has also been applied to Hi-C studies [85]. Nagano *et al.* have introduced a special pipeline for their single-cell protocol [110].

**Table 3:** Histone modifications and variants at enhancers

Histone modification/histone variant	Enzymes	Main observation	References
H3K4me1/2	KMT2C/2D (MLL3/4); KMT7 (SET7/9)	Active, intermediate and poised enhancers	[136–138]
H3K9ac	KAT2A/B (GCN5/PCAF)	Active enhancers	[115, 139]
H3K14ac	KAT2A/B (Gcn5/PCAF); KAT3A/3B (p300/CBP); KAT6A (MYST3)	Active enhancers	[139]
H3K27ac	KAT3A/3B (p300/CBP)	Active enhancers	[5]
H3K36me3	KMT3A (SET2)	Active enhancers	[140]
H3K56ac	KAT3A/3B (p300/CBP)	Active enhancers	[141]
H4K16ac	KAT8 (MOF)	Active enhancers	[142]
H3K9me3	KMT1E (SETDB1)	Poised enhancers	[140]
H3K27me3	KMT6 (EZH2)	Poised enhancers	[140, 143]
H2A.Z/H2A.Zac	KAT5 (TIP60)	Poised and active enhancers	[115, 144–146]
H3.3	–	Poised and active enhancers	[147, 148]

It is interesting to note the disagreement between ‘global' (e.g. Jin *et al.* [83]) and ‘local' methodologies (e.g. Rao *et al.* [56]) to determine significant interactions. Jin *et al.* normalize each observed interaction frequency against the average frequency of interactions with similar sequential distance. In parts of the genome, broader regions have generally high interaction frequency with other broader regions. When normalizing against global averages, Jin *et al.* may end up with several interactions between bins from two such regions. Rao *et al.* criticize such interactions for not being specific, and instead use a local normalization scheme that contrasts the interaction frequency between two bins with that of the broader local region around the two involved bins (denoted as the neighbourhood of a pixel in the terminology of Rao *et al.*). Even though Rao *et al.* [56] collected an order of magnitude more interactions than all previous Hi-C data sets combined (between 395 million and 1100 million contacts, depending on the cell and number of biological replicates), they only call 10 000 chromatin loops (compared with 100 000 loops in Sanyal *et al*., and 1 M in Jin *et al*.). Such disagreements are related to the fact that we have no means (based on 3C data only) to discern if an interaction is functional. By taking short-range interactions into consideration, we risk including a massive amount of non-functional interactions. But, by excluding them, we risk getting a bias towards only PEIs that are the result of discernible folding. One can thus think that future studies must take into account functional information before discarding non-atypical interactions.

The current situation of the field allows us to anticipate the development of ‘gold standard' data sets per cell type and time course. A ‘gold standard' has been called for in Hi-C analysis; however, as no such standard exists, we await better 3D simulations of chromatin [108]. Benchmark studies should also be developed to compare the different statistical methodologies, as well as new statistical methods that take functional information into account.

### Are chromatin interaction results reproducible?

Experimental reproducibility is an issue that has been marginally addressed in the field. Rao *et al.* report the number of peak annotation overlaps between a primary and a replicate Hi-C experiment, and their results show 5403 common interactions, with 2651 interactions unique to the primary experiment and 2081 interactions unique to the replicate [56]. Such agreement rates are a problem common to most chromatin-related data sets, and the most common way of addressing the issue has been merging the data from both replicates. That clearly shows that interaction datasets are catalogues of possible contacts in a population of cells that do not necessarily co-exist. Dynamic studies of PEIs [79, 109] and single-cell protocols [110] are needed to distinguish time-related or population-related variability from the variability derived from stochastic fluctuations at the molecular level. Such studies would help us to improve reproducibility of chromatin interaction maps.

### Is it possible to predict PEIs?

A PEI prediction methodology is a computational procedure that uses existing genomic data sets of specific cell types to predict PEIs of the same or a different cell type. Such an achievement would be important for at least three reasons: Firstly, genome segmentation methods and other enhancer prediction methodologies are producing many predicted enhancer regions, but they lack information regarding the associated target genes or promoters. Linking regulatory sequences to their target genes has been recognized as one of the main current challenges in transcriptional biology [20]. Secondly, this would help to link disease-related single-nucleotide polymorphisms (SNPs) occurring inside enhancer regions with their gene targets (an enhancer mutation can affect target gene regulation) [24, 111, 112]. Thirdly, we envision the possibility of using statistical approaches informed by biological knowledge to select relevant PEIs from Hi-C raw data.

To identify the gene corresponding to a predicted enhancer, the ‘nearest-promoter' was the first method applied. Variations of this method, such as the ‘nearest-expressed-gene' to an enhancer, or taking into account the limits established by insulator elements [113] have been reported. These methods are essentially inaccurate, as enhancers may be localized several kilobases or even megabases away from their promoters [114]. Ernst *et al.* and Thurman *et al.* introduced the first methods trying to predict PEIs based on the correlation to single epigenetic marks [115, 116]. The method by Ernst *et al.* predicts PEIs using the histone modification profile correlation, while Thurman *et al.* use DHS correlation. The first method is limited to the nearest candidate pairs within a 125 kbp distance, while the second one includes all candidate pairs within a 500 kbp distance.

Recently, more methods have appeared: The FANTOM5 consortium introduced a method for finding all expression correlations for all promoter–enhancer pairs in a 500 kb window [77]. The authors compared their results with the DHS correlations of Thurman *et al.* [116], and concluded that transcription is a better PEI predictor than chromatin accessibility (20.6% supported associations with CAGE expression correlation versus 4.3% with DHS). Another method is PreSTIGE [117], which predicts PEIs by correlating cell type-specific H3K4me marks (enhancer signals) with specific gene expression, across different cell types. The most recent method is IM-PET [118], which uses a Random Forest classifier that is based on four features: enhancer–promoter activity profile correlation, TF-promoter correlation, co-evolution of enhancer and promoter and distance between enhancer and promoter. The authors suggest much better results than all other attempts and suggest that PEIs have higher cell specificity than enhancers (49% of cell-specific PEIs versus 32% of cell-specific enhancers).

A pipeline combining chromatin interaction data processing with PEI prediction is High-throughput identification pipeline for promoter interacting enhancer element (HIPPIE) [119]. This platform starts from raw Hi-C reads, and then identifies high-confidence interacting fragments after mapping and evaluating polymerase chain reaction artefacts, restriction fragment size and GC-content. After that, HIPPIE predicts enhancers based on DHS, H3K4me1 and H3K27ac marks, and the low levels of H3K4me3 and H3K27me3, and predicts PEIs based on promoter–enhancer distance only. PEI prediction is the ultimate frontier of bioinformatics in the field of epigenetics. Methods that can be easily applied to different cell types and have a high predictive capacity are still needed.

## PEIs and disease

Chromatin interactions may offer an explanation to the effects of disease-associated non-coding variation [24, 114]. It is known that genomic variation in non-coding genomic regions (especially in enhancers) is linked to a number of diseases. Young and co-workers have postulated that most disease-related SNPs are found inside large clusters of enhancers known as ‘super-enhancers' (see also Box 2) [120]. However, the mechanisms that connect genome-wide association study (GWAS)-identified loci to their target genes and pathways have not been easy to explain until recent years, with the advent of PEI studies and the construction of chromatin interaction networks.

In Figure 3, we illustrate two examples where chromatin interactions have been used to explain the link between non-coding SNPs and disease [121, 122]. Here, we have used publicly available Hi-C data from human foetal lung fibroblasts [85] and the enhancer marks H3K4me3 and H3K27ac in different parts of the human brain and in foetal lung fibroblasts [123]. Davison *et al.* [121] reported a PEI-mediated mechanism for Type-1-Diabetes and Multiple Sclerosis and used it to predict a new disease candidate gene. The *CLEC16A* has been traditionally reported as the main candidate for several autoimmune diseases associated to the 16p13 region, such as Type-1-Diabetes, Systemic Lupus Erythematous and Multiple Sclerosis, because of the high number of disease-associated SNPs in intron 19. However, the authors show that this region in *CLEC16A* is enriched in enhancer marks, and they used 3C to map an interaction with the *DEXI* gene, whose function in autoimmune disease was not previously described. The combination of GWAS and 3C technologies allowed the authors to suggest *DEXI* as an autoimmune disease candidate gene, as well as provide a disease mechanism where the allelic variant disrupts a PEI between an enhancer in the intron of *CLEC16A* and *DEXI*. Using publicly available human data sets [85], we were able to visually identify enhancer marks in the region associated with the Type-1-Diabetes SNP (rs1208716) in the intron of *CLEC16A* in foetal lung fibroblasts (Figure 3A). One of the cell types that Davison *et al.* used for their 3C study was lung epithelial cells because *DEXI* is expressed in these cells. Therefore, we used publicly available Hi-C data in human foetal lung fibroblasts to identify interactions between *CLEC16A* and *DEXI* [85]. However, this data set has a limited resolution, and we could only show looping of intron 19 of *CLEC16A* to the first intron of *DEXI* gene, and not the promoter (Figure 3A). Recently, PEIs have also been proposed as an explanatory mechanism for obesity. Smemo *et al.* [122] found that a region rich in obesity-associated SNPs in the first intron of the *FTO* gene (which were also rich in enhancer-associated chromatin marks) displays a long-range interaction with the homeobox gene *IRX3* in both human and mouse. *IRX3* encodes for a TF highly expressed in the human brain, heart and lung and is important for control of body mass and composition. Based on their finding, the authors suggested a mechanism where allelic variants in the enhancer inside *FTO*’s intron may disrupt looping with the *IRX3* gene and affect *IRX3* expression, and not the expression of FTO [122]. Inspecting the publicly available human data sets [85, 123], we were able to visualize the findings of Nóbrega and co-workers. The region with Body Mass Index-associated SNPs in the intron of *FTO* shows looping with the *IRX3* gene in foetal lung fibroblasts and this region is enriched in enhancer marks in different brain tissues and lung fibroblasts (Figure 3B).

Both studies described here illustrate the power of combining 3C technology with GWAS studies in identifying candidate genes involved in disease. Several other studies linking PEI disruption to diseases have been published [124–127], including a few reviews. Horan and Ballard reviewed PEI disruptions in diseases including prostate, breast and colorectal cancers, facioscapulohumeral muscular dystrophy, neurological disorder and Rett syndrome [111]. A more recent review includes references to metabolic syndrome, coronary artery disease and human development [112]. A resource that collects and annotates genetic variants using chromosome conformation data is GWAS3D [128].

This review raises several different bioinformatics challenges related to PEI analysis. We anticipate the development of new bioinformatics tools, ‘gold standards', data processing methods and prediction methodologies that will have an important impact on PEIs in basic and biomedical research. Implementation of these will advance our understanding of the role of chromatin interactions in gene regulation, give us a better insight into PEIs across cell types, tissues and in different model organisms (at different time points or at different developmental stages) and provide mechanistic understanding of non-coding variation in disease.

Key Points

- Our understanding of the chromatin interaction network is becoming more complex. Distinguishing PEIs from all other chromatin interactions is a first challenge that demands both accurate enhancer detection methods and comprehensive genome annotation efforts.
- We present five models of chromatin looping. Whether transcriptional regulation is the cause or consequence of chromatin looping is not known. These models directly influence the bioinformatics approaches to PEI visualization, data processing and prediction, and point to the necessity of modelling of spatially co-localized groups of genomic regions (and not only linear sequences) to understand transcriptional regulation.
- Several chromatin interaction detection technologies are currently in use and under continuous development. These methods have made it possible to study genome-wide chromatin organization; however, they have limitations and call for use and development of alternative methods such as live-imaging studies in single cells. Generation of new data processing methodologies for 3C methods are needed to resolve discrepancies observed between FISH and 3C.
- There have been advances in PEI data representation, such as the creation of multiple file formats and visualization tools. We call for an agreement on standards. The existing tools also face some practical challenges, such as the increase in data resolution and dealing with the combination of long-range and short-range interactions.
- PEI prediction is the ultimate frontier of bioinformatics in the fields of transcription and epigenetics. A few methods are available, but high predictive capacity is needed. Future methods should go beyond chromatin accessibility and expression predictors and make use of chromatin organization. Such methods would help us to achieve another important goal, which is the identification of long-range functional targets of disease-related SNPs.
